# Supplementary figures and images for: Early Biofilm Formation on the Drain Tip after Total Knee Arthroplasty Is Not Associated with Prosthetic Joint Infection: A Pilot Prospective Case Series Study of a Single Center
Source: Healthcare (Basel). 2024 Jan 31;12(3):366. doi: 10.3390/healthcare12030366 (PMC10855896; doi:10.3390/healthcare12030366)

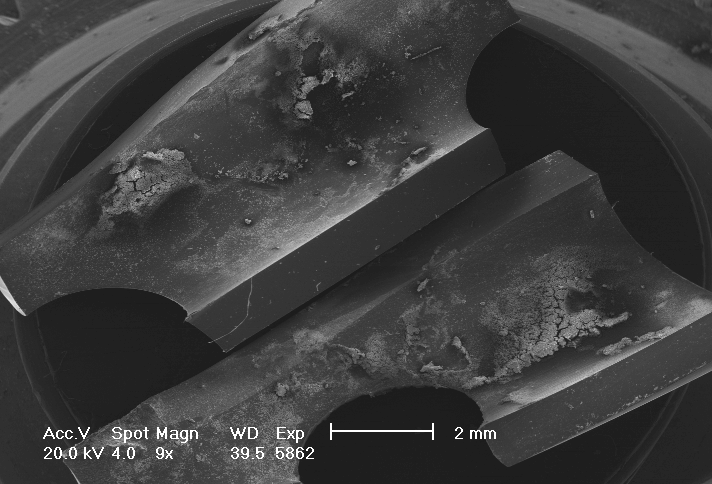

Supplement: Supplementary file 1 [file healthcare-12-00366-s001.zip › 586200 immagine panoramica catetere.TIF]

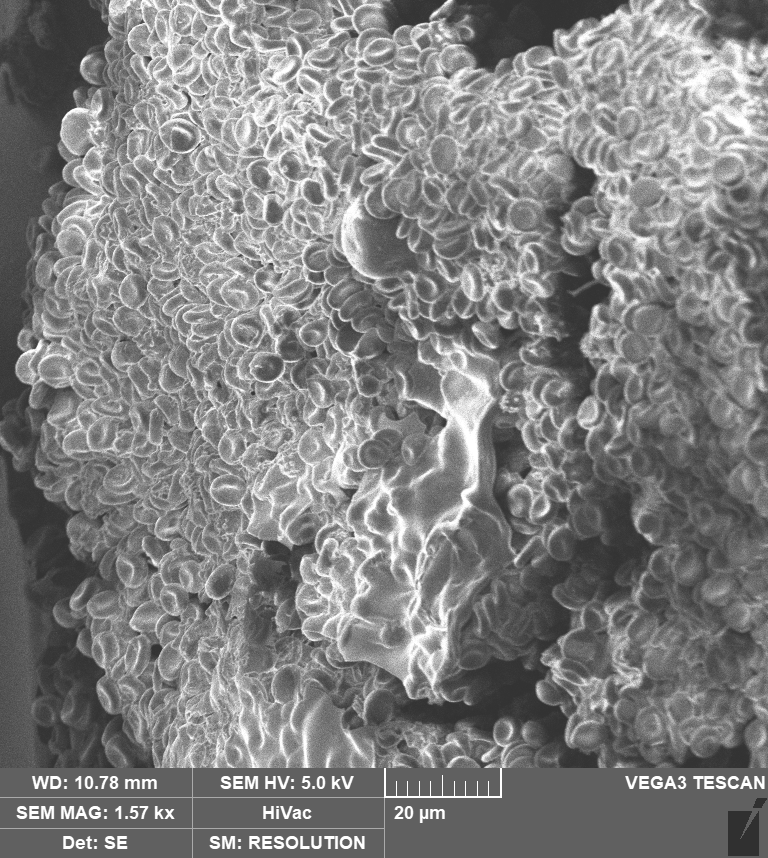

Supplement: Supplementary file 1 [file healthcare-12-00366-s001.zip › grado 0/5758 GR.tif]

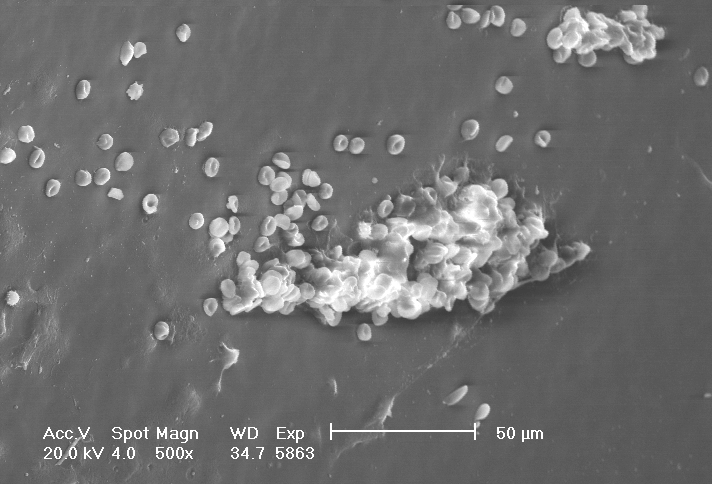

Supplement: Supplementary file 1 [file healthcare-12-00366-s001.zip › grado 0/586302.TIF]

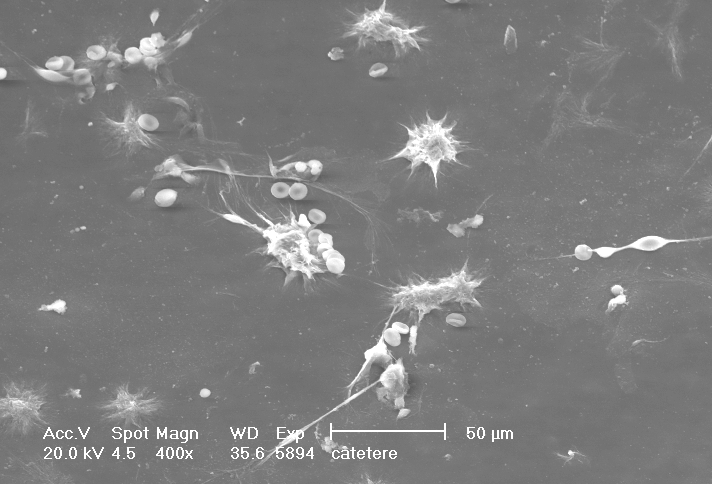

Supplement: Supplementary file 1 [file healthcare-12-00366-s001.zip › grado 0/589400 , coagulo ematico con gr e gb.TIF]

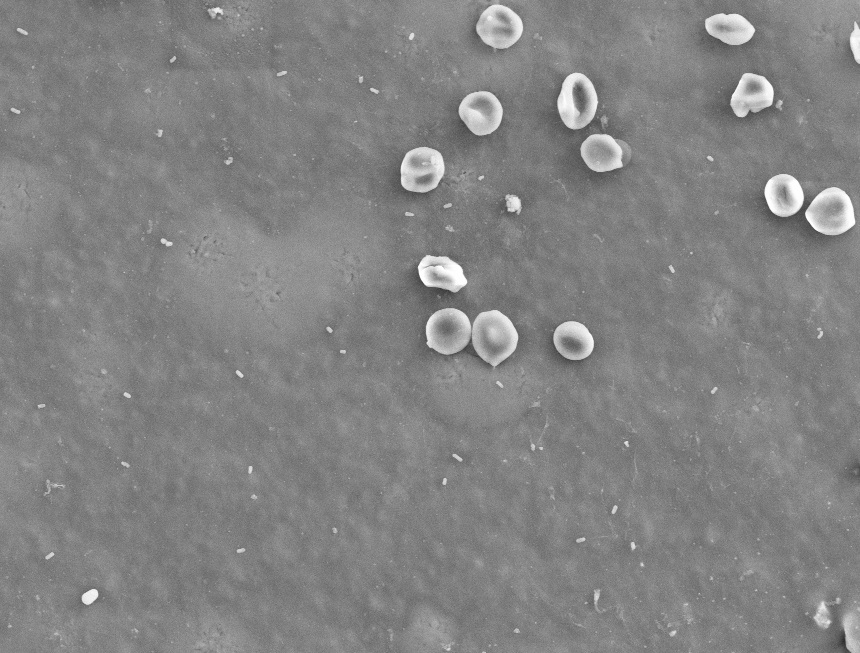

Supplement: Supplementary file 1 [file healthcare-12-00366-s001.zip › grado 1/5758_021.jpg]

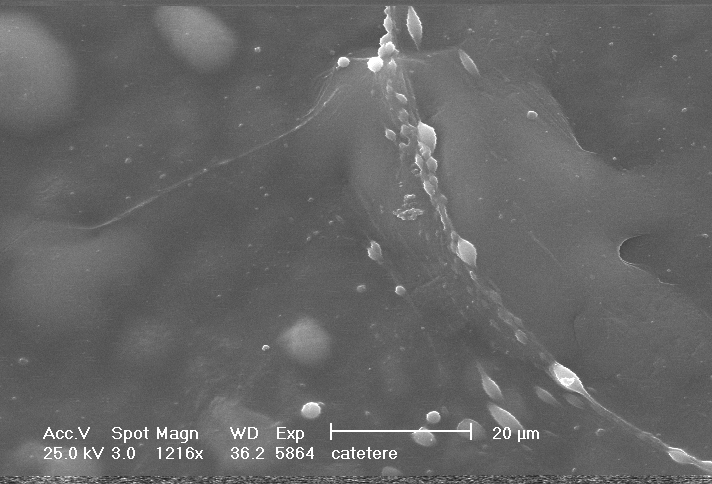

Supplement: Supplementary file 1 [file healthcare-12-00366-s001.zip › grado 1/5864006.TIF]

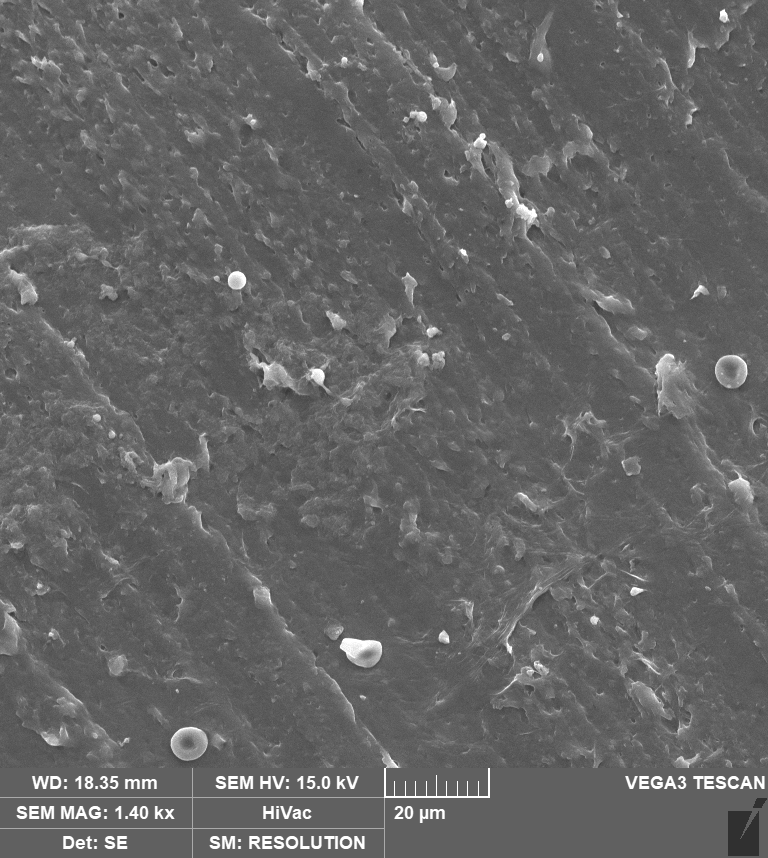

Supplement: Supplementary file 1 [file healthcare-12-00366-s001.zip › grado 1/5865_1 tappeto globuli bianchi.tif]

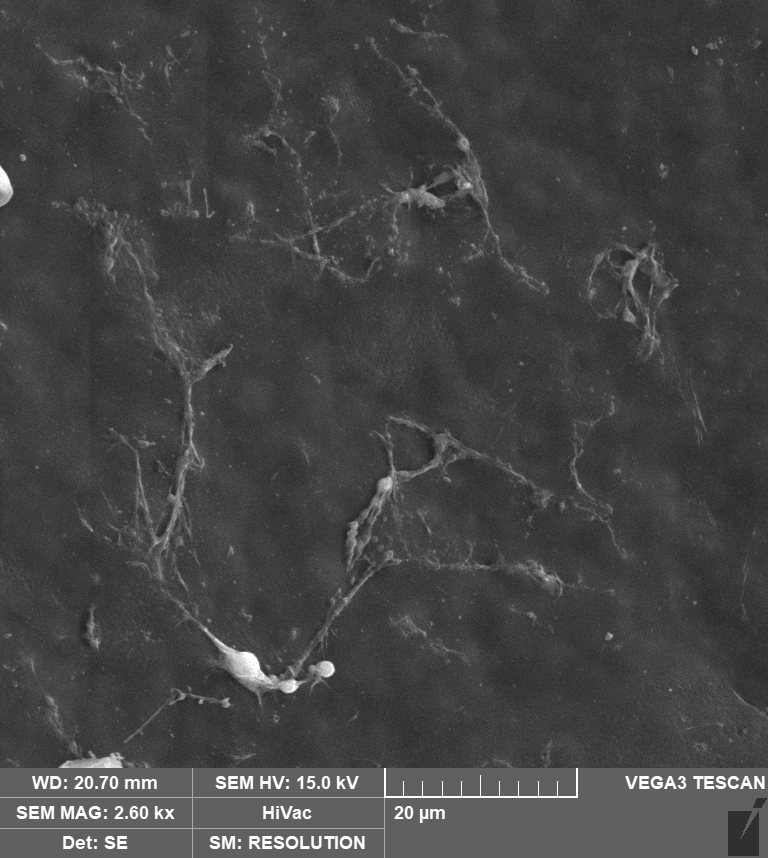

Supplement: Supplementary file 1 [file healthcare-12-00366-s001.zip › grado 1/5868_3.tif]

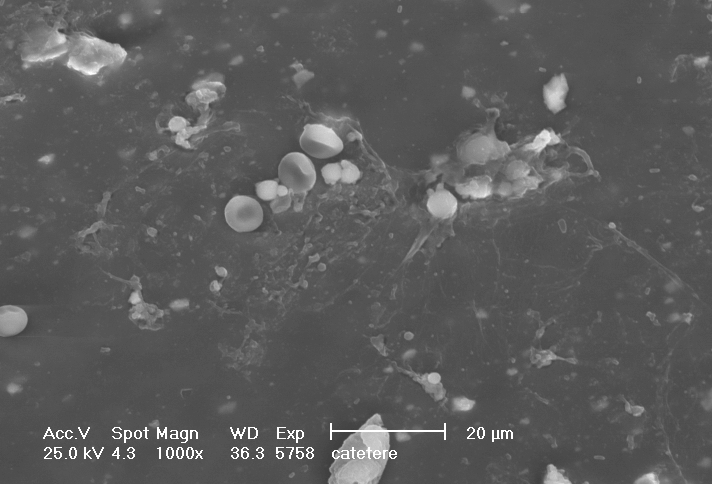

Supplement: Supplementary file 1 [file healthcare-12-00366-s001.zip › grado 2/5858003.TIF]

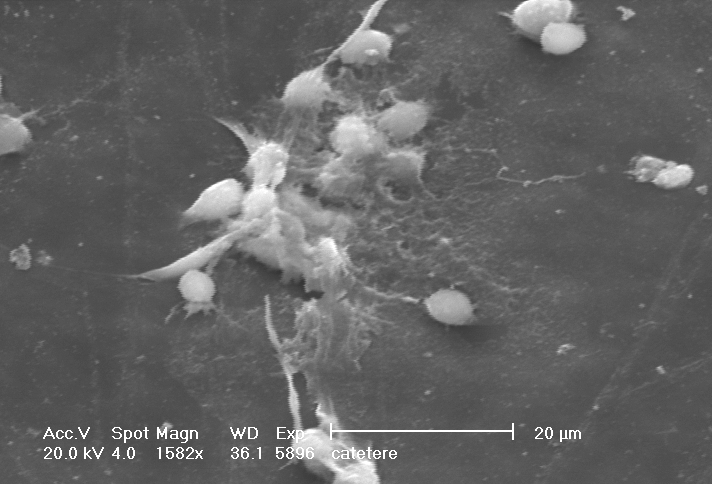

Supplement: Supplementary file 1 [file healthcare-12-00366-s001.zip › grado 2/589602.TIF]

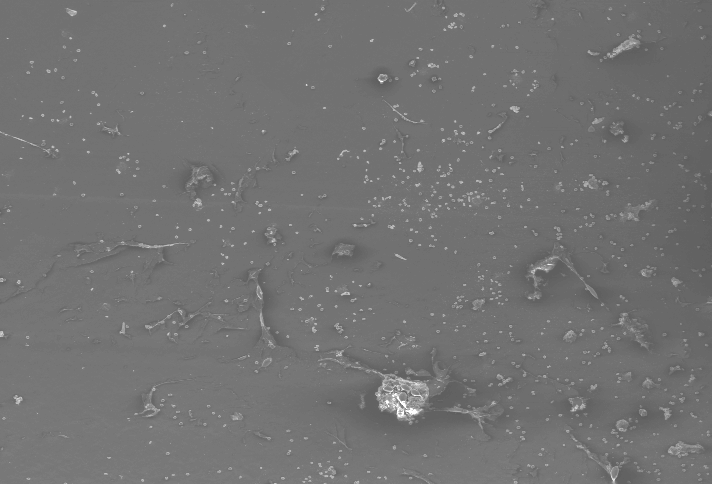

Supplement: Supplementary file 1 [file healthcare-12-00366-s001.zip › grado 3/5752002.TIF]

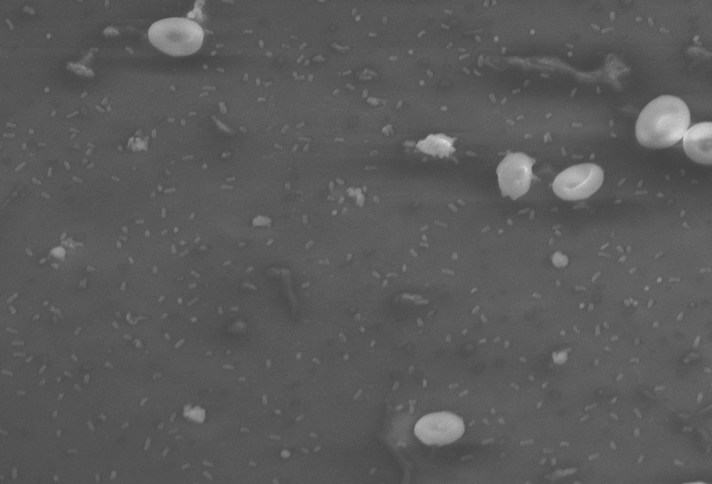

Supplement: Supplementary file 1 [file healthcare-12-00366-s001.zip › grado 3/5752005.TIF]

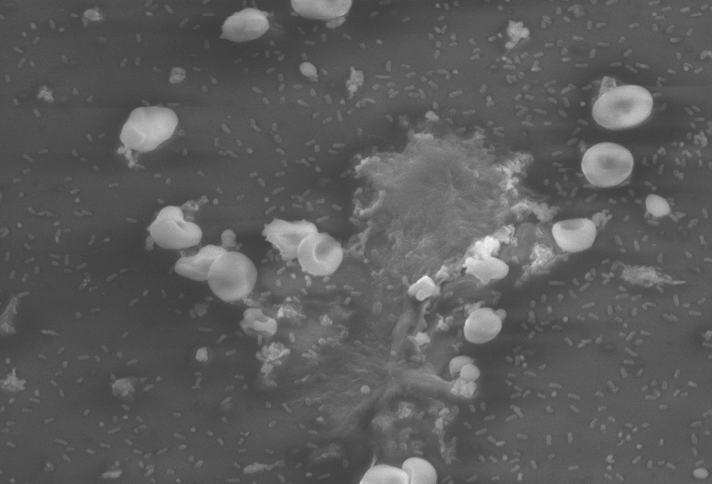

Supplement: Supplementary file 1 [file healthcare-12-00366-s001.zip › grado 3/5752007.TIF]

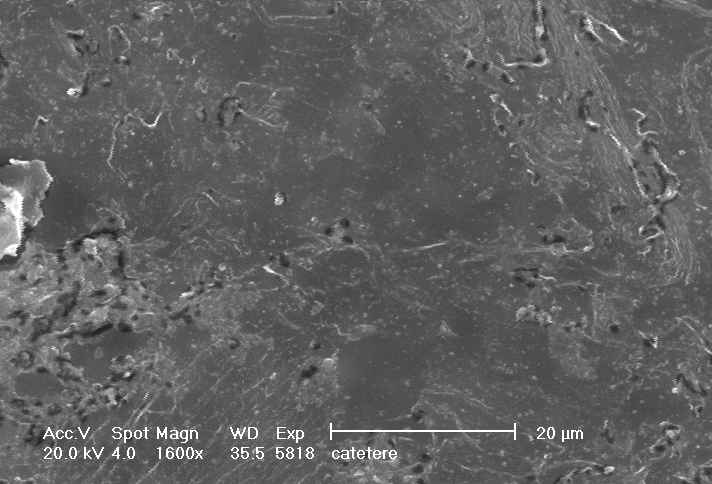

Supplement: Supplementary file 1 [file healthcare-12-00366-s001.zip › grado 3/581809.TIF]

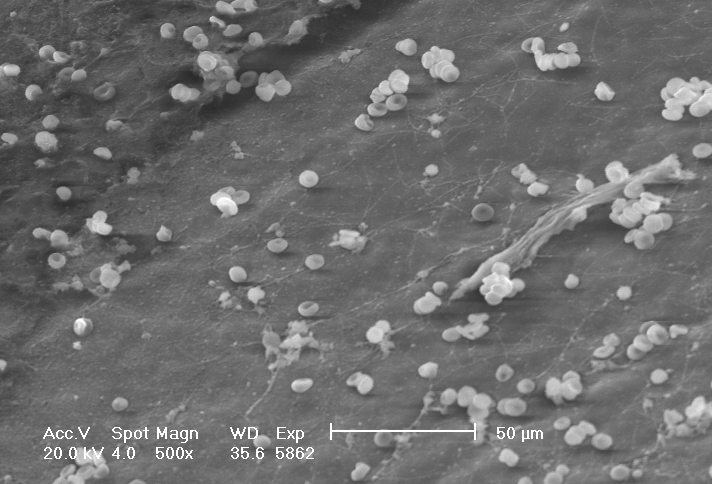

Supplement: Supplementary file 1 [file healthcare-12-00366-s001.zip › grado 3/586201.TIF]

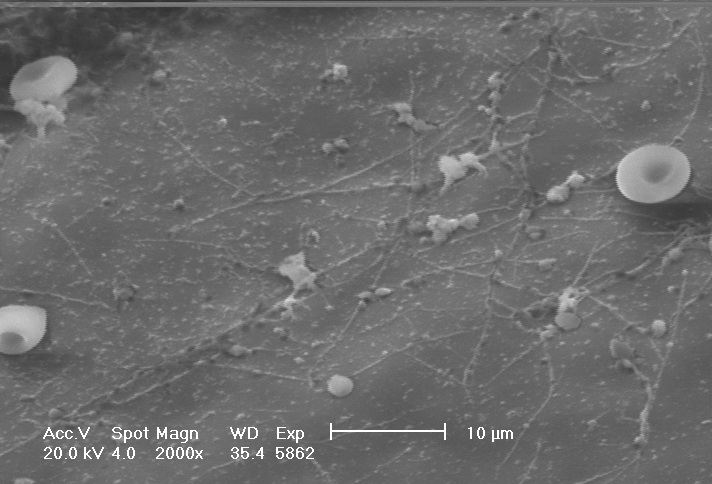

Supplement: Supplementary file 1 [file healthcare-12-00366-s001.zip › grado 3/586203.TIF]
